# Integrating a Fundus Camera with High-Frequency Ultrasound for Precise Ocular Lesion Assessment

**DOI:** 10.3390/bios14030127

**Published:** 2024-02-29

**Authors:** Alfa Rossi, Yushun Zeng, Mojtaba Rahimi, Taeyoon Son, Michael J. Heiferman, Chen Gong, Xin Sun, Mohammad Soleimani, Ali R. Djalilian, Mark S. Humayun, Qifa Zhou, Xincheng Yao

**Affiliations:** 1 Department of Biomedical Engineering, University of Illinois Chicago, Chicago, IL 60607, USA; arossi22@uic.edu (A.R.); mrahim8@uic.edu (M.R.); tyson08@uic.edu (T.S.); 2 Alfred E. Mann Department of Biomedical Engineering, Viterbi School of Engineering, University of Southern California, Los Angeles, CA 90089, USA; yushunze@usc.edu (Y.Z.); cgong841@usc.edu (C.G.); xsun7861@usc.edu (X.S.); humayun@usc.edu (M.S.H.); 3 USC Roski Eye Institute, Keck School of Medicine, University of Southern California, Los Angeles, CA 90033, USA; 4 Department of Ophthalmology and Visual Sciences, University of Illinois Chicago, Chicago, IL 60612, USA; mheif@uic.edu (M.J.H.); msolei2@uic.edu (M.S.); adjalili@uic.edu (A.R.D.); 5 USC Ginsburg Institute for Biomedical Therapeutics, University of Southern California, Los Angeles, CA 90033, USA

**Keywords:** transparent ultrasound transducer, widefield fundus camera, retinal imaging, ultrasound imaging

## Abstract

Ultrasound A-scan is an important tool for quantitative assessment of ocular lesions. However, its usability is limited by the difficulty of accurately localizing the ultrasound probe to a lesion of interest. In this study, a transparent LiNbO_3_ single crystal ultrasound transducer was fabricated, and integrated with a widefield fundus camera to guide the ultrasound local position. The electrical impedance, phase spectrum, pulse-echo performance, and optical transmission spectrum of the ultrasound transducer were validated. The novel fundus camera-guided ultrasound probe was tested for in vivo measurement of rat eyes. Anterior and posterior segments of the rat eye could be unambiguously differentiated with the fundus photography-guided ultrasound measurement. A model eye was also used to verify the imaging performance of the prototype device in the human eye. The prototype shows the potential of being used in the clinic to accurately measure the thickness and echogenicity of ocular lesions in vivo.

## 1. Introduction

Choroidal nevi are benign ocular tumors that are estimated to be present in 5% of adults over 40 years of age in the United States [1]. A small fraction of these lesions transforms into malignant lesions called uveal melanoma, which affect six non-Hispanic white individuals per million and at a lower rate in other races. However, due to being situated in the delicate structures of the eye, intraocular tumor biopsy and subsequent pathological examination are performed for diagnostic purposes only in exceptional cases where the ocular oncologist is unable to make a conclusive diagnosis after clinical examination and systemic evaluation [2]. After diagnosis of uveal melanoma, patients typically undergo enucleation or globe sparing radiation therapy that can severely affect vision [3]. Early diagnosis of these malignant lesions can avoid enucleation and significantly preserve the patient’s eyesight [4,5]. Furthermore, even with successful local disease control, 50% of patients diagnosed with uveal melanoma develop metastatic disease, with a high rate of subsequent mortality [3]. Therefore, early detection of malignancy is of paramount importance to preserve eyesight and improve survival in patients with uveal melanoma.

The evaluation of a suspected ocular tumor is performed with dilated fundus examination and multi-modal imaging to accurately identify benign and malignant lesions including choroidal nevi, uveal melanoma, choroidal metastasis, circumscribed choroidal hemangioma, choroidal osteoma, and retinoblastoma. With the advent of widefield and ultra-widefield fundus cameras [6,7,8], identifying suspected lesions even in the periphery of the retina is possible. Fundus images provide critical information, such as the size, location, color, presence of orange pigment, and presence of overlying drusen [2,9]. However, traditional fundus photography lacks depth-resolved information and typically shows superficial layer information which is not enough to determine other important characteristics of an ocular tumor. Spectral-domain optical coherence tomography with enhanced depth-resolved imaging (EDI SD-OCT) capability has shown promise in accurately illustrating the intrinsic optical characteristics of ocular lesions [10]. Despite being able to distinguish lesions from normal tissue, the low penetration capability of OCT limits its usage to only very thin lesions, typically less than 1 mm in thickness, especially in melanocytic lesions [11], whereas choroidal tumors can have a height of up to 10 mm [12]. Imaging techniques such as fluorescein angiography (FA), indocyanine green angiography (ICGA), and fundus autofluorescence (FAF) can provide valuable information such as changes in the retinal pigmented epithelium (RPE) overlying ocular lesions, tumor vasculature, and associated vascular leakage [1,2,13,14]. However, these imaging modalities do not provide sufficient information related to tumor depth, and their standalone diagnostic accuracy is relatively low.

Ultrasound imaging of the eye has been a longstanding imaging technique due to its excellent tissue penetration capability, non-invasive nature, and ability to differentiate tissues with different echogenicity [15,16]. Experimental research with different ultrasound transducer materials and frequencies is also gaining popularity for non-invasive imaging of the eye [17,18,19]. Usage of ultrasound is important in many subspecialities of ophthalmology including cataract surgery planning, glaucoma, vitreoretinal surgery, uveitis, oculoplastic procedures, and emergency medicine in addition to ocular oncology where it serves a critical purpose of diagnosing and monitoring ocular tumors, such as uveal melanoma [2,12,20]. For imaging the posterior retina, both a one-dimensional amplitude scan (A-scan) and a two-dimensional brightness scan (B-scan) are used [15]. The B-scan can be used to observe anterior and posterior segments of the ocular lesion as well as the axial and lateral dimensions of the eye. Although a one-dimensional vector A-scan could be derived from a B-scan image, its usability for quantitative measurement of lesion thickness is limited, as the logarithmic amplification used to generate the B-scan image prevents the quantitative analysis needed for accurate lesion echogenicity analysis [21]. Logarithmic amplification increases the dynamic range; however, it reduces the sensitivity, which further prevents the differentiation between tissues where the variation of echogenicity is minimal. Moreover, the focused beam used in the B-scan probe further complicates the process of computational echogenicity analysis of ocular lesions [21]. A specifically designed A-scan probe called standardized A-scan with non-focused parallel beam and S-shaped amplification was optimized for eye bio-microscopy [22,23] and has been an important part of many ophthalmology clinics ever since. Standardized A-scan has become the gold standard for calculating the apical height of ocular lesions, specifically because of its enhanced penetration capability and ability to differentiate the lesion border from the scleral tissue [15,16,21]. Several quantifiable parameters other than the apical height have been extracted out of A-scan ultrasounds, such as median internal reflectivity (%) of a lesion, heterogenous/homogenous echogenicity, number of internal reflectivity peaks inside a lesion, and sound attenuation pattern (angle ĸ) to facilitate an accurate clinical diagnosis [12,15,24]. The internal reflectivity and sound attenuation patterns are well established for differentiating choroidal melanoma from other lesions such as choroidal nevus, retinoblastoma, circumscribed choroidal hemangioma, and choroidal metastasis [12,24,25]. Given that treated choroidal melanomas show a considerable rise in median internal reflectivity, A-scan ultrasounds may also be useful in assessing the effectiveness of treatment [15].

Despite the promise, ultrasound A-scans come with a set of challenges. Precise guidance of the A-scan line towards the lesion requires an experienced operator, especially when the lesion is relatively small. Inaccurate guidance could lead to erroneous measurement of critical parameters such as apical height and internal reflectivity. Furthermore, the growth of benign lesions such as choroidal nevi could indicate a transformation towards malignant choroidal melanoma [26], and clinicians often observe the nevus size over a long period of time. However, monitoring the apical height from the exact same spot over a prolonged period is nearly impossible because of the absence of A-scan guidance. Therefore, accurate guidance of A-scan ultrasound probes is highly desirable for quantitative analysis, management, and documentation of ocular lesions.

The study explores the feasibility of combining fundus imaging with ultrasound measurements for ocular assessment, aiming to leverage fundus imaging as a reliable guide for ultrasound procedures. For the initial proof of concept, a widefield fundus camera was integrated with a transparent LiNbO_3_ single-crystal ultrasound transducer. In vivo experiments with rat eyes demonstrated the ability of the prototype to simultaneously gather fundus images and ocular ultrasound A-scans. The resultant A-scan clearly differentiated the anterior cornea, the eye lens, and the posterior retina. The optical performance of the imager was also examined with a model human eye and compared with the performance of the same imager but with a glass window replacing the transparent transducer. The resultant images show that the imager with a transparent transducer can clearly visualize the retina and can be readily implemented in clinical settings.

## 2. Materials and Methods

### 2.1. Design of the Transparent Ultrasound Probe

Based on the transparent application for the transducer, the transparent lithium niobate (LNO) single crystal was selected as the core component for the ultrasound transducer fabrication. The acoustic impedance of the LNO is 34 MRayl. Krimboltz, Leedom, and Mattaei (KLM) transducer equivalent circuit model-based modeling software PiezoCAD PRO 4.01 (Sonic Concepts, Inc., Woodinville, WA, USA) was applied to simulate and optimize the electrical impedance and pulse-echo performance of the designed transducer. To ensure transparency for light penetration, a colorless parylene film (Parylene C, Specialty Coating Systems Inc., Indianapolis, IN, USA, 2023) with acoustic impedance of 2.5 MRayl was chosen as the matching layer for acoustic impedance compensation, and a transparent epoxy (EPO-TEK 301, Epoxy Technology, Inc., Billerica, MA, USA, 2023) with acoustic impedance of 3.05 MRayl was determined as the backing layer for absorbing reflected ultrasound and penetrating optical source. The designed parameters of the transducer are summarized and listed in Table 1.

### 2.2. Fabrication of the Transparent Ultrasound Probe

**Figure 1:**
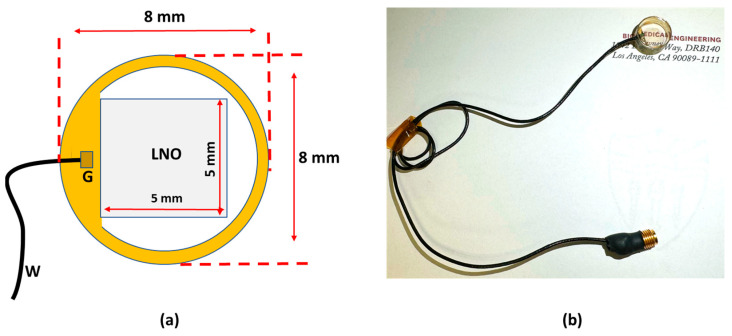
(**a**) The schematic diagram of the transparent transducer. (**b**) The fabricated transparent transducer with connections. G: ground; W: wire.

A 40 MHz transparent LNO disk wafer (Boston Piezo-Optics, Inc., Bellingham, MA, USA) with a size of 5 mm × 5 mm was purchased and manufactured for this study. Both sides of the LNO were sputtered indium tin oxide (ITO), a transparent electrode, by the sputtering system (NSC-3000 Sputter Coater, Nano-Master, Inc., Austin, TX, USA). Afterward, the LNO with electrodes was shielded by the brass housing and connected with one side of the double-shield coaxial cable, and the other side of the cable was connected with the Sub-Miniature version A (SMA) connector for ultrasound driving system connection. Degassed epoxy (EPO-TEK 301) was poured into the brass housing to form the backing layer. After 3 h of curing for the epoxy, the Au/Cr electrode (100 nm/50 nm) was sputtered to conduct the ITO edge of the LNO and brass housing for ground connection. Finally, the parylene film was coated on the surface of the LNO as a matching layer and water-proof layer; then, the fabrication of the transparent transducer was finished and was further characterized and tested. Figure 1a illustrates the schematic diagram of the transparent ultrasound probe. The transducer has 5 mm × 5 mm clear penetration window. Figure 1b shows the finished cylinder-type transparent ultrasound transducer. The letters printed on the background could easily be read, further indicating the transparency of the transducer.

### 2.3. Testing the Performance of the Transparent Ultrasound Probe

The electrical impedance and phase spectrum of the fabricated transparent transducer were measured via an impedance analyzer (HP 4294A, Agilent Technologies Inc., Santa Clara, CA, USA). The pulse-echo performance of the transparent transducer was measured via the setup detailed in previous work [27]. The transducer was immersed into the water tank, and the quartz plate was applied as a reflector for reflecting generated ultrasound. The transducer excited by pulser-receiver (Panametrics 5900PR, Olympus NDT Inc., Waltham, MA, USA) with 1 µJ energy per pulse transmitted and received the ultrasound. The axial resolution *(R_axial_*) of the transducer can be calculated using Equation (1) [27].

(1) $$R_{axial}=\frac{\lambda }{2BW}$$

where *λ* is the wavelength of the ultrasound in water (37 MHz–40 μm), and *BW* is the −6 dB fractional bandwidth. Thus, *R_axial_* can be estimated. The optical transmission spectrum of the transparent transducer was calculated by passing the light from a broadband LED (MBB1L3, Thorlabs Inc., Newton, NJ, USA) through the transducer and analyzing the transmitted light using a UV-VIS spectrometer (USB400, Ocean Optics Inc., Dunedin, FL, USA).

### 2.4. Experimental Setup

**Figure 2:**
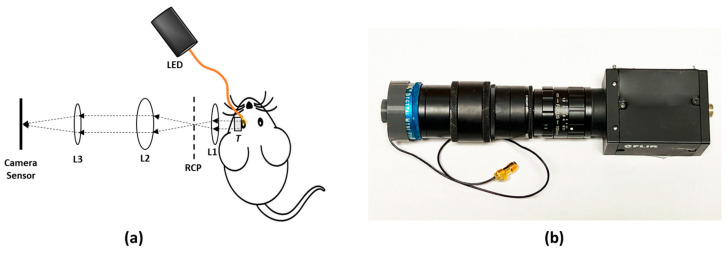
(**a**) The schematic illustration of the experimental setup. (**b**) Photographic illustration of the imaging system. RCP: Retina conjugate plane.

Figure 2a illustrates the schematic diagram of the experimental setup for in vivo retinal imaging of rat eyes. The light source is a visible light LED (M530L4, Thorlabs Inc., Newton, NJ, USA) with a center wavelength of 530 nm and a full-width half maximum (FWHM) of 35 nm. For albino rats, the contrast of the retinal vasculature is poor when visualized with red and NIR light, and the UV portion of the spectrum is heavily attenuated by the ocular lens. Therefore, the choice of wavelength in the green portion of the spectrum was made as it shows the retinal vessels with excellent contrast [28]. An optical fiber (MHP550L02, Thorlabs Inc., Newton, NJ, USA) coupled the LED light to the eyelid of the rat and the end of the fiber was fixed to the eyelid with tape. The optical power at the tip of the fiber was measured to be 5 mW. The imaging system consisted of the transparent ultrasound probe as the frontal element, followed by an ophthalmic lens (L1) (f = 11 mm) (Volk Digital Series Wide Field lens, Volk, Mentor, OH, USA), a relay lens (L2) (f = −50 mm, LC1715, Thorlabs Inc., Newton, NJ, USA), and a camera lens (L3) (12 fixed focal length lens, 33-303, Edmund Optics Inc., Barrington, NJ, USA). The working distance of L1 was chosen with the thickness of the transparent ultrasound probe in mind, so that, when the transducer touches the cornea properly, a retinal image is created by L1 at the retina conjugate plane (RCP) and this image is relayed to the camera sensor (GS3-U3-41S4M-C, FLIR Integrated Imaging Solutions Inc., Richmond, BC, Canada) by L2 and L3. Figure 2b shows a photographic illustration of the imaging system.

Furthermore, we investigated how the transparency of the ultrasound probe might affect image quality during human eye imaging. In order to do that, we imaged an eye model (OEMI-7, Ocular Instruments, Bellevue, Washington, DC, USA) first with the proposed imaging system. Then the ultrasound transducer was replaced with a glass window (WG10530, Thorlabs Inc., Newton, NJ, USA). The glass window is similar in thickness to the ultrasound transducer, but optically neutral. Ultrasound signal was generated and received by a pulser receiver (Panametrics 5900PR, Olympus NDT Inc., Waltham, MA, USA) and a custom-made MATLAB script was created to visualize and perform adequate filtering on the signal. A custom-made LABVIEW interface was created to control image acquisition.

### 2.5. Animal Preparation

The Association of Research in Vision and Ophthalmology’s guidelines for the ethical use of animals in ophthalmic and visual science research were followed in all aspects of the experimental protocols and related animal care. The related experimental protocol was approved by the University of Illinois Chicago’s (UIC) Animal Care Committee. The study employed a sample of eleven-month-old male Sprague Dawley rats (*N* = 2). The rats were acquired from Charles River Laboratories (Wilmington, MA, USA) and housed within UIC’s Biology Resource Lab. After the rats were anesthetized, the pupil was dilated using a drop of 1% tropicamide ophthalmic solution. A lubricant ocular gel (GenTeal, Alcon Laboratories Inc., Fort Worth, TX, USA) was applied to the whole anterior eye to keep the eye surface hydrated throughout the experiment. The eye gel also served as a coupling medium between the transparent ultrasound probe and the eye to ensure the propagation of the ultrasound waves. An animal holder was used to keep the rat steady and keep the head of the rat immobilized. The animal holder facilitated five degrees of freedom position controls.

## 3. Results

**Figure 3:**
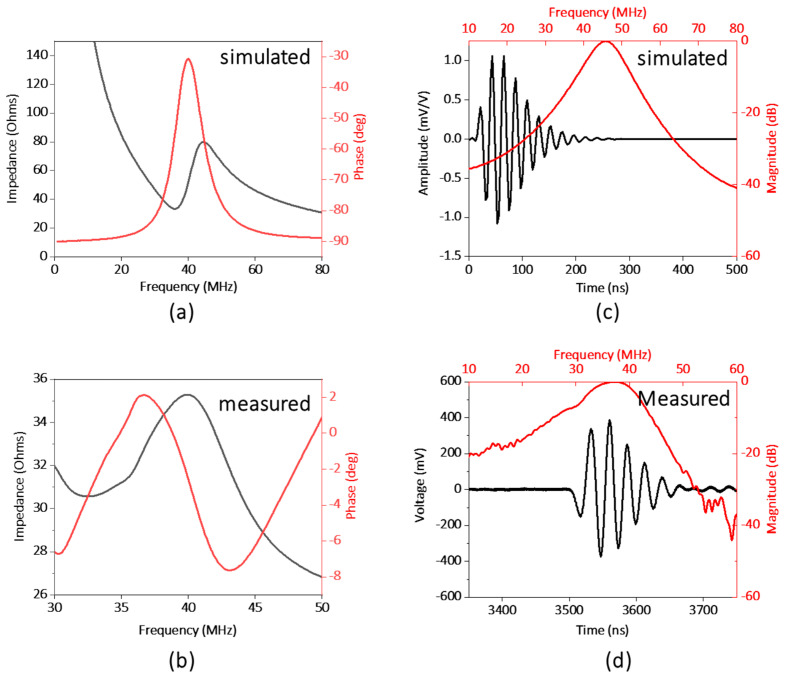
(**a**) Simulated and (**b**) measured electrical impedance and phase spectrum of the transducer. (**c**) Simulated and (**d**) measured pulse−echo performance of the transducer.

Figure 3a,b illustrates the electrical impedance and phase spectrum of the fabricated transparent transducer. The resonant frequency (*f_r_*) and anti-resonant frequency (*f_a_*) were located as 32 and 41 MHz, respectively, which is close to the simulation results. Hence, the effective electromechanical coupling coefficient (*k_eff_*) of the transducer, describing the conversion efficiency between electrical energy and mechanical energy, is calculated to be 0.62 via Equation (2) [27].

(2) $$k_{eff}=\sqrt{1-\frac{f_{r}^{2}}{f_{a}^{2}}}$$

The electric impedance magnitude of the fabricated transducer is in the range of 30 to 40 Ω around the resonance frequency of the transducer, approximately closing to match the ideal electrical impedance of 50 Ω. The pulse-echo performance of the transducer is illustrated in Figure 3c,d. The result shows the central frequency of the transducer as 37 MHz, and the −6 db fractional bandwidth as 30%, which are in good agreement with the simulation results (40 MHz). And based on Equation (1), *R_axial_* can be further estimated as 66 μm.

**Figure 4:**
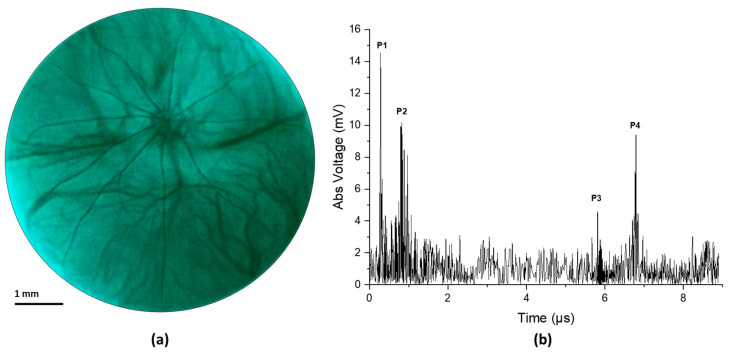
(**a**) Fundus image taken from in vivo imaging on a rat eye. (**b**) Ultrasound pulse-echo data gathered from the same rat eye.

Figure 4 illustrates the result from an in vivo experiment in a rat eye. Figure 4a shows the fundus image and Figure 4b shows the corresponding ultrasound pulse-echo image. The acquired pulse echo signal constitutes both positive and negative voltage. However, to ensure similarity with the clinical ocular ultrasound pulse-echo signal, we illustrated the absolute voltage instead of the real voltage. It is evident from Figure 4a that the retinal and choroidal vessels as well as the optic nerve of the rat retina are clearly imaged in the fundus photograph. The ultrasound data show four distinct peaks corresponding to four anatomical locations inside the eye, namely the cornea (P1), the anterior capsule of the crystalline lens (P2), the posterior capsule of the crystalline lens (P3), and the retina (P4). The axial length of the eye could be approximated from the time interval between the corneal peak and the retinal peak, given that we know the speed of sound in the eye. The speed of sound in aqueous humor and vitreous humor is around 1532 m/s, whereas the speed of sound inside the ocular lens is around 1641 m/s [29]. For conservative estimation, we assume that the speed of sound inside the whole eye is 1532 m/s. We find the axial length of the eye using the following equation,

(3) $$D=\frac{\Delta t\times v}{2}$$

where Δt is the time interval between the corneal peak and the retinal peak and v is the speed of ultrasound. The axial length was calculated to be 4.902 mm, which is close to the axial length reported in other studies on a similar strain of rats measured via OCT [30]. The slight mismatch in axial length from the reported value could be attributed to the fact that the lens encompasses more than 60% of the volume of the eye in rats [31]. So, our simple approximation of a constant velocity would not be sufficient for calculating the axial length correctly. However, for the human eye, the lens is small relative to the whole eye, so even a simple approximation could give us a nearly accurate result. Furthermore, the velocity of sound in different human eye tissues and their interfaces is well characterized [32]. 

**Figure 5:**
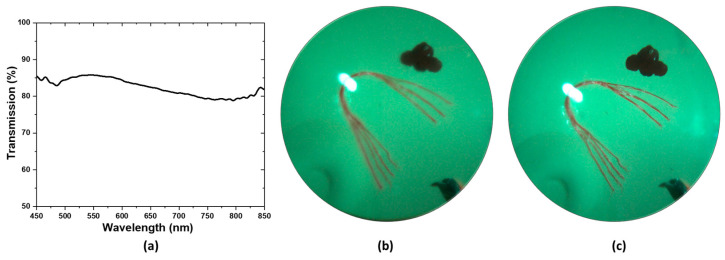
(**a**) Transmission spectrum of the transparent transducer (**b**) Image of the retina of an eye model taken with the prototype. (**c**) Image of the same model eye taken after replacing the ultrasound transducer in the prototype with a glass window.

Figure 5a illustrates the optical transmission spectrum of the ultrasound transducer. The optical transmission is above 80% in the blue, green, and red portion of the spectrum and goes slightly below 80% at the NIR portion of the spectrum. The effect of the transparent transducer on the imaging performance of the system is illustrated in Figure 5b,c. Figure 5b illustrates an image of the retina of an eye model taken with the proposed prototype. Figure 5c illustrates an image of the retina of the same eye model taken after replacing the transparent transducer with an optically neutral glass window. The glass window was chosen in such a way that it did not change the magnification of imaging and only the difference in imaging performance would be highlighted. The structures present in the eye model, namely the blood vessels, the optic nerve head, and the pigmented ocular lesions on the superior and inferior retina could be easily identified in both images. The mean RGB intensity of the images taken with the transparent transducer and the glass window are 121 and 135, showing the illumination efficiency of the device is not severely affected by the transparent transducer. The ocular vessel branches present in the model eye are clearly seen in both images. However, the smaller vessels that constitute the vessel branches could be differentiated in the image taken with the glass window, whereas the image taken with the transparent transducer does not resolve the smaller vessels in the vessel branches clearly. That being said, the quality of the image taken with the transparent transducer should be sufficient to accurately guide the ultrasound signal to ocular lesions, which is the goal of the prototype.

## 4. Discussion

In summary, we demonstrated the feasibility of using a fundus camera to guide the local position of an ultrasound transducer to detect pulse-echo signals from the posterior segment of the eye. A high-frequency transparent ultrasound transducer was used to facilitate simultaneous fundus and ultrasound imaging. In vivo testing was performed on rats with our prototype and simultaneous fundus image and ultrasound A-scan were gathered. Furthermore, the optical performance and image quality of the prototype on the human eye were examined with a model eye. In addition to providing objective guidance for ultrasound imaging, the fundus image would also provide valuable information such as the presence of orange pigment or drusen overlying an ocular tumor, which are important features in the diagnosis of melanocytic choroidal tumors [1,26,33].

For this feasibility study, we utilized a high-frequency ultrasound probe with a center frequency of 40 MHz to differentiate the cornea, the ocular lens, and the retina clearly in the rat eye. The rat retina is much thinner than the human retina [34], and needs a high-frequency ultrasound signal to differentiate different ocular structures properly. The −6 dB axial resolution of the system was calculated to be 66 μm, which is reasonable for imaging the rat eye. On the other hand, the attenuation of ultrasound waves significantly increases with the operating frequency, reducing the penetration depth. The smaller axial length of a rat eye (around 7 mm) [29] helps to acquire signals from the retina with reasonable quality. In the human eye, the attenuation coefficients for the cornea, lens, and aqueous and vitreous humor are 0.78, 1.19, and 0.10 dB/cm/MHz, respectively [35,36]. At high frequencies, significant signal attenuation occurs when traversing the eye’s large globe (axial length 22–24 mm [37]), rendering the acquisition of meaningful retinal signals unfeasible [20]. Therefore, we were not able to conduct ultrasound measurements from the human eye with this preliminary light-ultrasound prototype. Ultrasound probes used in ophthalmology clinics generally have a center frequency between 7 MHz and 20 MHz [16] to compensate for the attenuation to enhance the penetration depth. We are currently pursuing a transparent ultrasound transducer with a frequency similar to the clinical ophthalmic ultrasound systems for future studies of human eyes. 

The ultrasound transducer used in this study has a 5 mm × 5 mm square-shaped aperture. For this unfocused transducer, as the structures depicted in Figure 4b reside within the near field zone of the ultrasound probe [38], the lateral resolution is estimated to be equal to the beam width, which is approximately close to the aperture size of the transducer. Figure 4a illustrates a retinal region measuring 6.5 mm in diameter, while the ultrasound signal in Figure 4b is derived from a central 5 mm × 5 mm window of the retinal image. The large beam width means that the echo signal received by the transducer comes from a larger area inside the eye, which in turn makes the signal noise prone. Notably, the signal coming from the posterior of the crystalline lens (peak P3 in Figure 4b) has a relatively weaker signal strength. This could be attributed to the large beam width and the acute curvature of the posterior section of the rat lens as a portion of the beam reaches the border while the rest is still traveling inside the lens, creating an averaging effect. Although the feasibility of guiding the ultrasound beam is validated with our prototype, for human usage, the frequency and the aperture size of the ultrasound probe need to be modified to achieve natural focusing on the retina plane (24 mm from the cornea) with lateral resolution comparable to the basal diameter of small ocular lesions (around 1.25 mm [1]).

An eye model was used to verify the effect that the transparent ultrasound probe had on the image quality (Figure 5b,c). For the actual human eye, factors such as pupil size and eye movement could affect the result. Therefore, the model eye was a better choice for accurate comparison. To deliver the light inside the model eye, a small hole was drilled 5 mm away from the cornea of the model eye and a small optical fiber was inserted which delivered the illumination inside the eye. 

For in vivo testing in rat eyes, the retina was illuminated through the eyelid, which is contrary to the conventional method of both delivering the illumination and receiving the retinal reflection through the pupil. This method has recently been proposed to acquire widefield retinal images even with limited pupil size in human eyes [39]. However, the added complexity of having a separate illumination path would increase the complexity of fundus image-guided ultrasound acquisition in patients. Miniaturized indirect illumination-based fundus imagers have shown promise in acquiring non-mydriatic widefield fundus images without the complexity of conventional pupillary ring illumination or a separate illumination system. A simple solution to the pupil limitation without requiring a separate illumination system is provided by miniature indirect illumination, which offers a different approach towards the development of widefield portable fundus cameras [40,41]. In our future prototype, we plan to include this illumination method to construct a compact, portable, widefield fundus image-guided ultrasound probe to accurately extract ultrasound information from ocular lesions in human patients. 

## 5. Conclusions

A fundus image-guided ultrasound probe was demonstrated for accurate measurement of ultrasound pulse-echo signals from the posterior segment of the eye. In vivo imaging of rat eyes demonstrated the feasibility of capturing retinal images and ultrasound measurements simultaneously. The optical performance and the image quality of the prototype were further validated using a model eye to demonstrate potential usability in humans.

## 6. Patents

Pending patent application for multimodal fundus-ultrasound eye imager.

## Figures and Tables

**Table 1:** Design parameters of the transparent ultrasound transducer.

Parameter	Value
Center frequency	40 MHz
Surface area	5 mm × 5 mm
Lithium niobate (LNO) thickness	70 µm
Matching layer (Parylene) thickness	10 µm
Backing layer (Epo-Tek 301) thickness	5 mm

## Data Availability

The data presented in this study are available within the article.
